# Oral Mucosal Melanoma In Situ Manifesting as Multiple Gingival Macules Around a Dental Implant–Based Prosthesis: A Case Report

**DOI:** 10.1155/crid/1357786

**Published:** 2026-08-29

**Authors:** Athina Tosiou, Eleni-Marina Kalogirou, Dimitrios Goutas, Nikolaos Katsoulas, Konstantinos I. Tosios

**Affiliations:** ^1^ Oral Medicine Private Practice, Athens, Greece; ^2^ Faculty of Health Sciences, Metropolitan College, Athens, Greece, mitropolitiko.edu.gr; ^3^ Istomedica Anatomic Pathology Laboratory, Athens, Greece; ^4^ Department of Oral Pathology and Medicine, and Hospital Dentistry, National and Kapodistrian University of Athens, Athens, Greece, uoa.gr

**Keywords:** case reports, dental implants, gingival neoplasms, melanoma, melanoma-specific antigens

## Abstract

**Introduction:**

Oral malignant melanoma in situ is a rare lesion that may precede the development of invasive oral malignant melanoma. We report an unusual presentation of a rare oral malignant melanoma in situ as multiple gingival macules around a dental implant–supported prosthesis, posing a significant diagnostic challenge.

**Method:**

A 68‐year‐old Caucasian male presented with two black macules on the buccal gingiva of a dental implant and pontic of the implant‐based prosthesis; they were asymmetric, with discrete borders, smooth surface, and homogenous color. Two small areas of gray discoloration were also seen on the palatal gingiva. The provisional diagnosis was postinflammatory pigmentation.

**Results:**

Following excision of all lesions, an oral mucosal melanoma in situ was diagnosed. A watch‐and‐wait approach with a 3‐month follow‐up was decided. Twelve months later, the lesion recurred in the subpontic area, and wide excision on clinically normal margins of the gingival mucosa to the level of periosteum was performed.

**Conclusions:**

This case emphasizes that peri‐implant pigmentation should not automatically be attributed to inflammatory or implant‐related causes. Early biopsy remains essential because oral mucosal melanoma in situ may present deceptively and can recur despite initial complete excision.

## 1. Introduction

Primary oral mucosal melanoma (OMM) is a rare aggressive melanocytic malignancy with poor prognosis. The age‐standardized rate is less than 0.01 cases per 100,000 person‐years globally [1], and it accounts for approximately 0.5%–1% of all melanomas [2, 3] or 0.26%–0.5% of all oral malignancies [1, 3].

A systematic review of 2230 cases reported that there was no considerable gender difference; the mean age of the patients at diagnosis was 58.2 years, and there was a strong predilection for localization on the palatal and gingival mucosa [4]. The clinical appearance of OMM is diverse [5] as it may present as a macule, plaque, or tumor, occasionally ulcerated, with a variably dark color, and irregular borders and surface [3]. It may be nonpigmented (amelanotic) and very rarely multifocal or with satellite lesions [6–8]. An early OMM is typically asymptomatic and may go unnoticed by the patient until it reaches a more advanced stage, as it arises in oral sites that are not readily accessible to self‐examination and may lack pigmentation [5]. Pain or other neurological symptoms, hemorrhage, bone destruction with teeth mobility, or ill‐fitting of removable dentures are late events in the disease course [3].

The etiology and pathogenesis of OMM remain unknown, and no risk factor has been identified up to date [3, 4, 9]. Mechanical stress to the maxilla due to mastication, occlusion, and deglutition has been attributed to the more common occurrence of melanoma in the oral and respiratory mucosa [9]. The genetic profile of OMM differs markedly from that of cutaneous melanoma, but also among different studies. Overall, *KIT* mutations are common, whereas the classic to cutaneous melanoma *BRAF^V600E^* and *NRAS* mutations are rare [10, 11]. The biological behavior of OMM is also different from that of cutaneous melanoma. A recent systematic review and meta‐analysis reported nodal positivity of 73% and an average 5‐year survival of 34% [12].

Histologically, OMM is classified as in situ, invasive, and invasive with in situ component [6]. The in situ (noninvasive) OMM (OMMIS) shows a melanocytic proliferation confined to the epithelium and the epithelial–connective tissue interface, without invasion into the underlying connective tissue [6]. OMMIS is considered rare [13] and may precede an invasive OMM [6].

We report an unusual presentation of a rare OMMIS as multiple gingival macules around a dental implant–supported prosthesis, posing a significant diagnostic challenge.

## 2. Case Report

A 68‐year‐old Caucasian male was referred by his dentist for diagnosis and management of pigmented macules on the gingiva of dental crowns #23 (Federation Dentaire Internationale tooth numbering system) and #25. Teeth #23, #24, and #26 had been restored with titanium dental implants and the latter two were abutments in a fixed prosthesis with #25 as a pontic. The implants were placed 36 months before presentation in an “apparently normal” alveolar ridge mucosa, according to the patient′s dentist, and the prosthesis was placed 6 months later. Twenty‐two months later, the dentist noticed that the gingival mucosa adjacent to #23 and #25 was “red,” and 8 months later that the color had changed to black. At this time, the patient was referred for oral mucosal examination. The patient was medicated with tamsulosin for benign hypertrophy of the prostate and quit smoking approximately 11 years before presentation. There was no personal or family history of melanoma.

**Figure 1:**
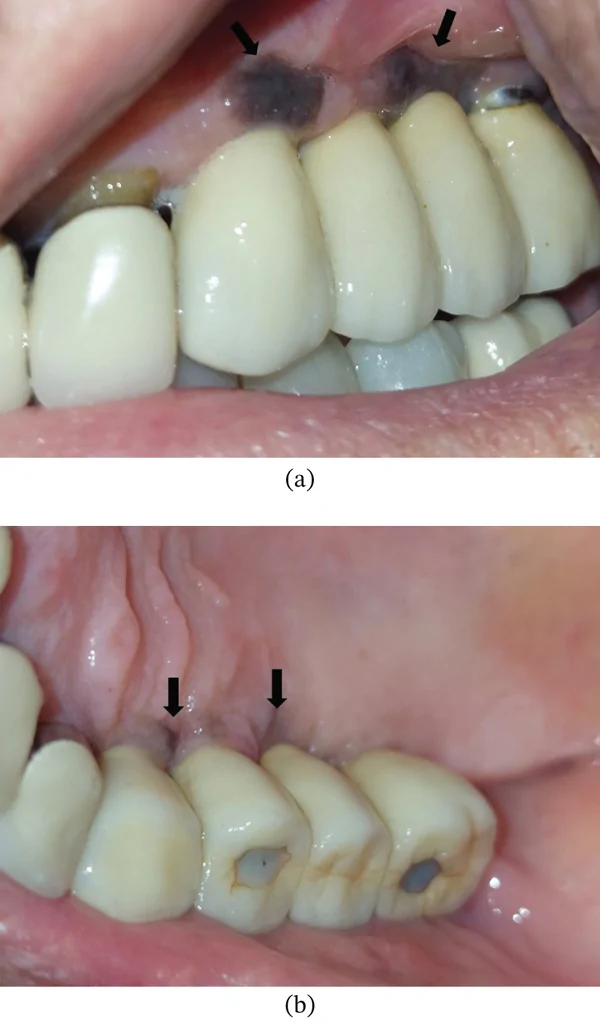
Clinical presentation. (a) Circumscribed black macules with discrete borders, smooth surface, and homogenous color on the buccal gingiva of dental implant #23 (FDI tooth numbering system) and pontic #25 (arrows). (b) Two small areas of gray discoloration on the palatal gingiva distal to implant #23 and proximal to pontic #25 (arrows).

Clinical examination at presentation showed two black macules on the buccal gingiva of dental implant #23 and pontic #25 (Figure 1a). They were asymmetric, with discrete borders, smooth surface and homogenous color, and measured 0.4 × 0.4 cm and 0.6 × 0.3 cm, respectively. On the palatal gingiva distal to crown #23 and proximal to pontic #25, two areas of gray discoloration, measuring < 0.2 cm each, were also seen (Figure 1b, arrows). The implants did not show bleeding on probing. The rest of the oral mucosa and head and neck examination was normal.

The multiplicity of the lesions, their peri‐implant distribution, and the history of preceding local inflammation posed a diagnostic challenge and led to the provisional diagnosis of reactive postinflammatory pigmentation. The macules from the buccal gingival mucosa and the two small areas of gray discoloration from the palatal gingival mucosa were totally excised separately under local infiltration anesthesia without suturing.

**Figure 2:**
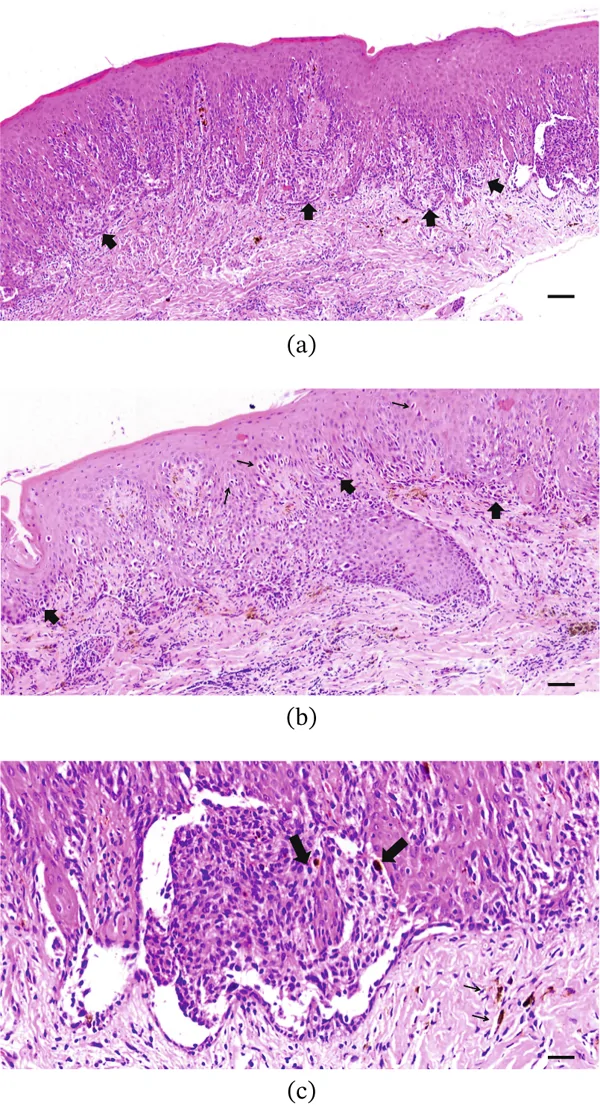
Histological examination. Parakeratinized oral mucosa showing melanocytes proliferating (a) as medium‐ to large‐sized melanocytic nests (arrows) and (b) in a lentiginous pattern (thick arrows) along the mucosal surface. Notice the pagetoid spread of single melanocytes or small aggregates/nests of melanocytes throughout all levels of the epithelium (thin arrows). (c) Neoplastic melanocytes containing fine melanin granules (arrows). (Hematoxylin and eosin stain, scale bars a = 100*μ*m, b = 50*μ*m, and c = 25*μ*m).

The specimens were fixed in 10% buffered formalin and submitted for histopathological examination. Grossly, two flat, brown‐colored tissue specimens measuring 0.5 × 0.2 cm and 0.8 × 0.3 cm, and two partly gray‐colored specimens measuring 0.4 × 0.2 cm and 0.3 × 0.2 cm were observed. Microscopic examination of 5‐*μ*m thick hematoxylin and eosin–stained sections disclosed that all four tissue fragments consisted of parakeratinized oral mucosa showing the development of a junctional melanocytic lesion with marked architectural dysplasia and significant cytologic atypia. Specifically, melanocytes appeared to proliferate as medium‐ to large‐sized melanocytic nests (Figure 2a) and in a lentiginous pattern along the mucosal surface (Figure 2b). Multiple foci of pagetoid spread were present, consisting either of single melanocytes or of small aggregates/nests distributed throughout all levels of the epithelium. The neoplastic melanocytes showed moderate nuclear atypia and pleomorphism, a conspicuous nucleolus, and lightly pigmented cytoplasm containing fine melanin granules (Figure 2c). In the superficial lamina propria, there were moderately dense lymphoplasmacytic inflammatory infiltrates, moderate stromal fibrosis, and numerous scattered melanophages (Figure 2c).

**Figure 3:**
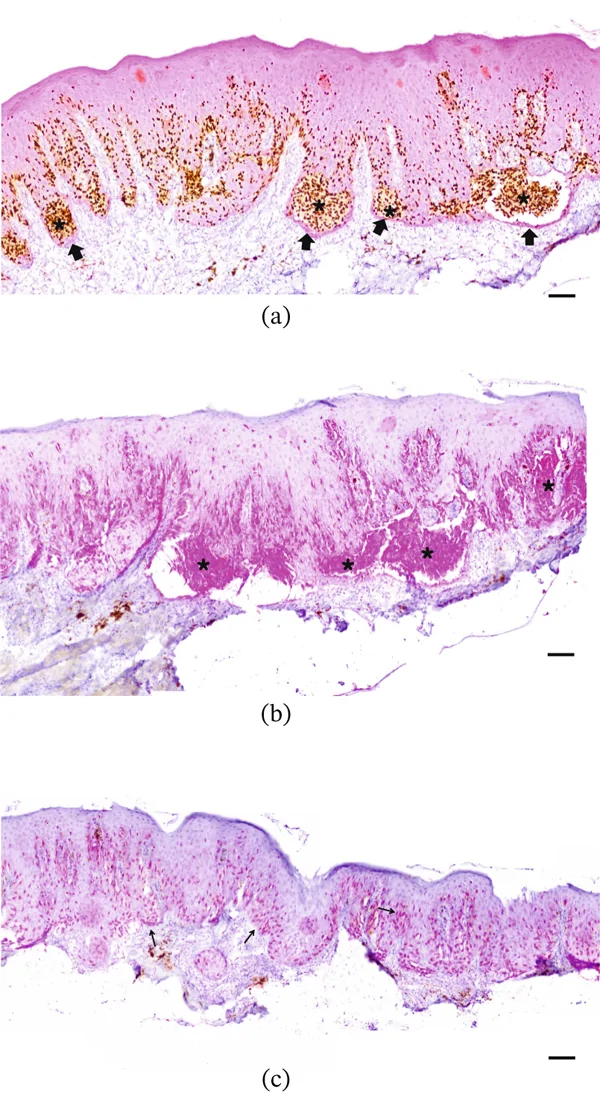
Immunohistochemical examination. (a) Double staining for CK14 (cytoplasmic, violet) and SOX10 (nuclear, brown) confirms the intraepithelial location of the SOX10‐positive melanocytes, as epithelium intervenes between melanocytic nests (asterisks) and connective tissue stroma (arrows). (b) Melanocytes in nests show intense cytoplasmic (violet) Melan‐A positivity (asterisks). (c) Intense nuclear (violet) PRAME immunoreactivity (Avidin–biotin–peroxidase immunohistochemistry, scale bars = 100 *μ*m; specimen from the buccal attached gingiva of implant #23).

Immunohistochemically, double staining for CK14 (Clone LL002 1:200, Leica Biosystems, Deer Park, Illinois, United States)/SOX10 (Clone IHC010 1:150, GenomeMe, Richmond, Canada) confirmed the intraepithelial location of the melanocytes (Figure 3a) that were intensely positive for Melan‐A (Clone A103 1:300, BioSB, Santa Barbara, California, United States) (Figure 3b) and PRAME (score 4, > 75%; ERP20330 1:4000, Abcam, Cambridge, United Kingdom) (Figure 3c). The morphologic and immunohistochemical findings were consistent with an OMMIS involving the entire extent of the submitted tissue fragments.

Postoperative healing was uneventful. Removal of the prosthesis and wide excision of the involved area was recommended to the patient, on the basis that the identical immunomorphologic features of all samples were more consistent with an OMMIS spreading in a pagetoid/lentiginous growth pattern, rather with clonally independent multiple primary (multifocal) lesions. Moreover, it could not be excluded either that the normal appearing intervening mucosa was not involved by the OMMIS, nor that an invasive focus was present in an unsampled area.

The patient was referred to a melanoma oncology unit for further evaluation and management. Following removal of the prosthesis, clinical examination of the alveolar ridge mucosa reported no residual pigmentation. Contrast‐enhanced computed tomography (CT) of the head and neck, and whole‐body positron emission tomography (PET) showed no evidence of maxillary bone invasion or regional or distant metastatic disease. Routine laboratory investigations were unremarkable. In the absence of evidence of residual or metastatic disease, as well as limited evidence regarding management of OMMIS, the melanoma team recommended a watch‐and‐wait with clinical follow‐up every 3 months.

**Figure 4:**
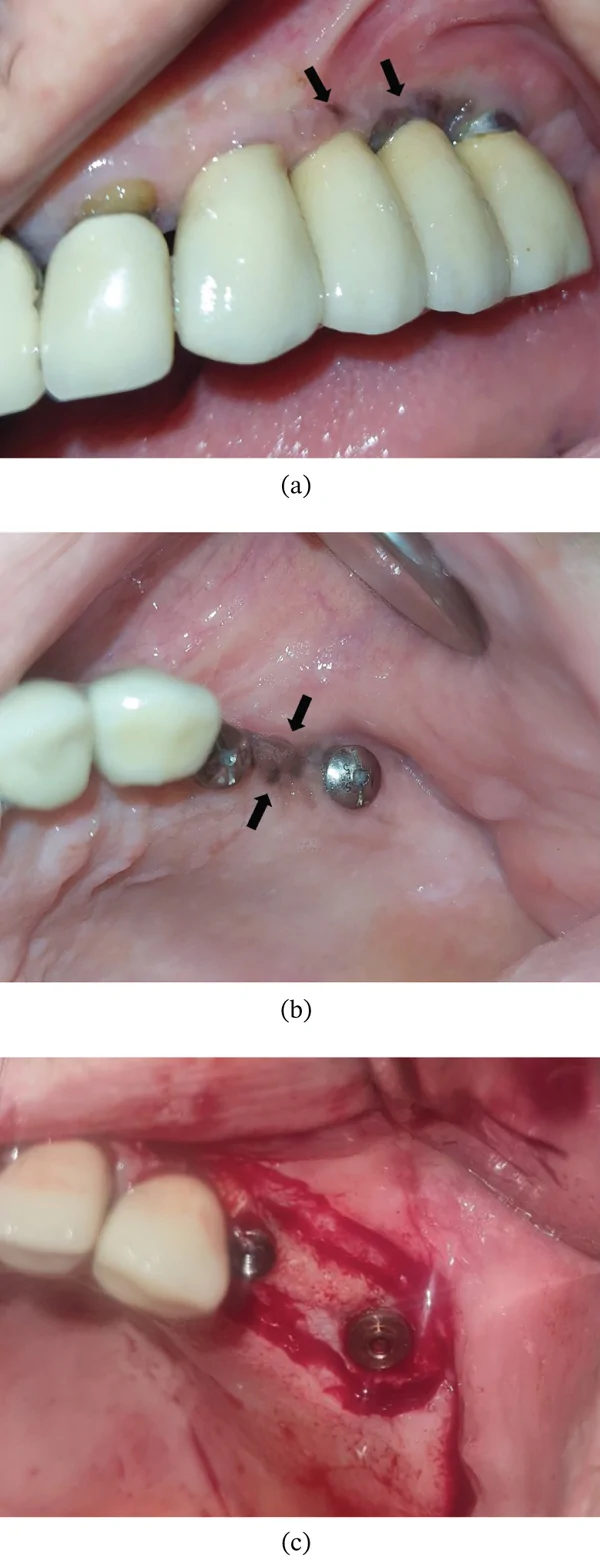
Clinical presentation of recurrent lesion. (a) Black macules at the buccal gingiva of implant #24 and pontic #25 (arrows), (b) occupying most of the subpontic mucosa (arrows). (c) Immediate postsurgical view of the area following excision on clinically normal margins of the gingival mucosa to the level of periosteum.

Twelve months following the initial diagnosis, black macules were noticed at the buccal gingiva of implant #24 and pontic #25 (Figure 4a). Removal of the prosthesis revealed that those lesions represented parts of a pigmented macule with indistinct borders and uneven color, occupying most of the subpontic mucosa (Figure 4b). The lesion was completely excised, and histopathological examination revealed a malignant junctional melanocytic lesion composed of lentiginously arranged melanocytes, with rare formation of medium‐sized nests. The melanocytes exhibited cytological and immunohistochemical features comparable to those observed in the first biopsy. They did not invade the lamina propria but extended close to (< 0.5 mm) one surgical margin. These findings were consistent with recurrent OMMIS. CT of the head and neck, PET scan, and laboratory work‐up performed in the melanoma oncology unit were negative for local infiltration or metastatic disease. This time, complete re‐excision was suggested to the patient. Under local infiltration anesthesia the gingival mucosa proximal to #23 to distal to #26 was excised on clinically normal margins, up to the level of periosteum (Figure 4c). Histopathological examination disclosed a small residual focus of OMMIS and confirmed negative radial margins (> 0.6 cm). Postoperative healing was uneventful. The patient is in a 3‐month follow‐up and was disease‐free on his first visit.

**Figure 5:**
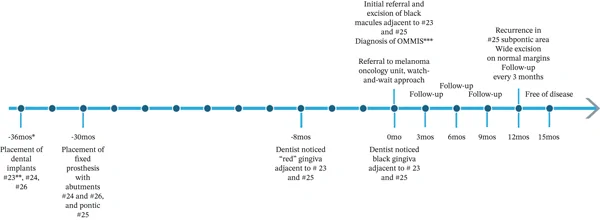
Timeline that summarizes the chronological milestones of the case. Abbreviations:  ^∗^mo(s): month(s);  ^∗∗^Federation Dentaire Internationale tooth numbering system;  ^∗∗∗^OMMIS: oral mucosal melanoma in situ.

The chronological milestones of the case are summarized in Figure 5.

## 3. Discussion

In the present case, an OMMIS that manifested as multiple macules on the gingival mucosa following the placement of titanium dental implant–based prosthesis was clinically misdiagnosed as a reactive pigmented lesion. A biopsy allowed prompt diagnosis, but following a watch‐and‐wait approach, there was recurrence of the lesion, finally necessitating wide surgical excision.

OMMIS appears to be a rare type of OMM, but an exact estimation of incidence is not possible as cases may have been reported under descriptive terms, such as premalignant melanosis, atypical melanocytic hyperplasia or proliferation, premalignant melanocytic dysplasia, superficial spreading melanoma [6, 14]. Barker et al. [6] diagnosed six cases of OMMIS among 50 cases of OMM; Prasad et al. [8], two cases among 31 OMMS; and Smith et al. [7], one case among 46 OMMs. A recent systematic review tabulated 28 documented cases of OMMIS and reported an additional case [13]. For three of those cases involving the lips, it is unclear whether the primary site of the lesion was on the skin or lip mucosa [15–17], whereas another case [18] most probably represented multifocal intraepithelial spread of a recurrent OMM. At least three cases of OMMIS have been documented [8, 19, 20] since this review. Overall, most patients with OMMIS were adult men, and most lesions were located on the palatal or gingival mucosa, with none of them close to implants [13, 20]. Multiple macules were described in five cases [13, 14, 19, 21, 22] and could be considered as either multifocal satellite lesions originating from the same primary one [1, 6, 7] or multicentric lesions developing due to field cancerization [23]. The clinical features of our patient are consistent with those previously reported, whereas the proximity of the macules to one another and their identical immunomorphologic features are more consistent with an OMMIS that has extended in a pagetoid/lentiginous manner.

The present case posed a diagnostic challenge, due to the unusual association of the macules with the dental implant–based prosthesis and multiplicity. Bluish‐gray discoloration is not uncommon around dental implants in patients with a thin gingival phenotype, as the metallic gray color of the implant or abutment can shine through the soft tissues [24]. In this case, however, the macules either did not conform to the implant′s shape or were in the pontic area. Titanium particles are commonly found in the soft and hard tissues around titanium dental implants, possibly due to electrochemical corrosion or mechanical wear and friction during implant placement, initiating a localized foreign body reaction termed metallosis [25, 26]. Titanium particles were identified in 41% of 153 oral mucosa specimens adjacent to implant cover screws [25] and 52.9% of 68 oral lesions associated with dental implants [27], but neither study described clinically apparent mucosal discoloration. Two cases of dark discoloration of the attached gingiva overlying titanium dental implants connected with zirconia abutments were attributed to wear of the titanium from zirconia and embedding of metal particles in the soft tissues [28]. However, the composition of the pigmented material was not identified, whereas a mechanism for the diffusion of the material to the gingiva was not suggested. Therefore, an association of the macules with the metals of the dental implant and prosthesis was discarded.

Amalgam tattoos are the commonest acquired pigmented oral lesions and may present as multiple macules, mostly on the alveolar mucosa and gingiva, in areas of postextraction sockets or around dental crowns [29]. However, they are usually gray shaded, whereas both the oral surgeon that placed the dental implants and the dentist that restored them did not notice any discoloration in the area during those procedures. Oral melanotic macules are the most common melanocytic lesions of the oral mucosa, but show a predilection for the vermilion border of the lips and are typically solitary; multiplicity is usually associated with systemic medications, systemic diseases, or inherited syndromes [29], not relevant to this patient′s medical history. Melanoacanthoma may present with multiple macules that expand rapidly [29], but unlike our case, it usually affects black women in the 3rd–4th decade of life, shows a predilection for the buccal mucosa and is very rare on the gingiva [29, 30]. Multiple oral melanoacanthomas and oral melanotic macules were described on the hard palate of a patient 1 month following implants placement [31], but in contrast to the present report, they were not located close to the implants.

OMM has a predilection for the palatal and gingival mucosa [4, 13], but very rarely may present with multiple macules [6, 7]. The value of the classical ABCDE criteria applied for the diagnosis of early cutaneous melanoma (i.e., asymmetry, border irregularity, color variegation, diameter > 6 mm, and evolution) in OMM has been questioned [32]. In the present case, the lesions were rather asymmetric, and one of them measured 0.6 cm in diameter, but they exhibited regular borders and a homogeneous color, whereas prompt excision precluded the assessment of evolution over time. In contrast, the clinical features of the recurrent lesion fulfilled all ABCDE criteria. Furthermore, association of OMM with dental implants has been described in just two cases [33, 34]. Both cases presented as pigmented tumors in males, aged 78 years [33] and 59 years [34], 8 years and 3 years following implants′ placement, respectively. Interestingly, the latter case was clinically diagnosed as pigmentation from the metal of the crown. Due to the very rare occurrence of OMM with multiple macules or in association with dental implants, this diagnosis was not considered prior to biopsy. In contrast, as in our case discoloration was preceded by “red” gingiva, a finding consistent with inflammation, posttraumatic/postinflammatory pigmentation was considered the provisional diagnosis. Posttraumatic/post inflammatory pigmentation may develop into an area of trauma or ulceration and, as melanin is deposited in the connective tissues or phagocytized be melanophages, it persists even after resolution of the initial lesion [5, 29]. However, as diagnosis of such lesions can be established only through clinicopathologic correlation, biopsy was mandatory.

The pathogenesis of OMM remains unknown. It has been hypothesized that trauma and mechanical stress on the oral mucosa may be involved by enhancing mitogen‐activated protein kinase signaling in melanocytes [9]. Therefore, it was suggested that ill‐fitting dental implants can underly OMM development [34]. In our case, however, OMMIS involved the subpontic mucosa, an area not expected to be subjected to direct trauma or mechanical stress by the implant‐based prosthesis.

OMMIS prognosis is obscure and there are no specific guidelines for its management, mostly due to its rarity. It is considered curable if diagnosed and treated promptly [35], but it may recur or develop into an invasive OMM and metastasize [13, 20, 36]. Among the 28 cases reviewed by Jasper et al. [13], eight recurred and two metastasized to cervical lymph nodes. In the study of Yip et al. [20], recurrence rate was 30% at an average follow‐up period of 35 months.

Wide surgical excision with 1‐cm clear margins is the recommended treatment [35], particularly in the presence of satellite lesions [6, 14]. Other treatment modalities, including tooth extraction, maxillectomy, elective node dissection, and adjuvant radiotherapy and chemotherapy have also been reported [13, 20]. In the present case, an initial watch‐and‐wait (active surveillance) approach was adopted, based on evidence from cutaneous melanoma in situ that some lesions may exhibit indolent biological behavior with a low risk of progression to invasive melanoma [37]. Therefore, wide surgical excision was considered unnecessary, as potential morbidity was not associated with any potential survival benefit for the patient [37, 38]. Following recurrence approximately 12 months after diagnosis, however, wide mucoperiosteal excision on clinically unaffected margins was judged mandatory. As an in situ phase may precede the development of invasive melanoma by many years [6], close, long‐term follow‐up is guaranteed.

This case emphasizes that peri‐implant pigmentation should not automatically be attributed to inflammatory or implant‐related causes. Early biopsy remains essential because OMMIS may present deceptively and can recur despite initial complete excision.

## Author Contributions

A.T. and E‐M.K. contributed to writing—original draft; D.G. and K.I.T. contributed to writing—review and editing; A.T., E‐M.K., and N.K. contributed to investigation; E‐M.K. and N.K. contributed to visualization.

## Funding

This study was supported by the Hellenic Academic Libraries Link (10.13039/100018996).

## Disclosure

All authors have read and approved the final version of the manuscript. K.I.T. had full access to all the data in this study and takes complete responsibility for the integrity of the data and the accuracy of the data analysis.

## Ethics Statement

The guidelines of the Helsinki Declaration were followed in this single anonymized case report. The patient gave his written informed consent for the use of his personal data and photographs for scientific purposes.

## Conflicts of Interest

The authors declare no conflicts of interest.

## Patient Perspective

The patient experienced initial anxiety after the unexpected diagnosis, but clear communication from the multidisciplinary team helped build confidence throughout treatment. Despite multiple procedures, temporary prosthesis removal, and short‐term pain with difficulties in eating and speaking, he understood the importance of treatment and long‐term follow‐up. Favorable pathology and follow‐up results progressively reduced anxiety, and the patient emphasized the value of prompt evaluation of suspicious oral lesions and adherence to follow‐up recommendations.

## Data Availability

The data that supports the findings of this study are available from the corresponding author upon reasonable request.
